# Isolation of *Streptomyces* inhibiting multiple-phytopathogenic fungi and characterization of lucensomycin biosynthetic gene cluster

**DOI:** 10.1038/s41598-024-57888-0

**Published:** 2024-04-02

**Authors:** Heung-Soon Park, Seung-Hoon Kang, Si-Sun Choi, Eung-Soo Kim

**Affiliations:** Department of Biological Sciences and Bioengineering, Inha University, Incheon, 22212 Republic of Korea

**Keywords:** *Streptomyces*, Antifungal activity, Phytopathogenic fungicide, Polyene macrolide, Genome mining, Biochemistry, Microbiology, Molecular biology

## Abstract

Soil microorganisms with diverse bioactive compounds such as *Streptomyces* are appreciated as valuable resources for the discovery of eco-friendly fungicides. This study isolated a novel *Streptomyces* from soil samples collected in the organic green tea fields in South Korea. The isolation process involved antifungal activity screening around 2400 culture extracts, revealing a strain designated as *S. collinus* Inha504 with remarkable antifungal activity against diverse phytopathogenic fungi. *S. collinus* Inha504 not only inhibited seven phytopathogenic fungi including *Fusarium oxysporum* and *Aspergillus niger* in bioassays and but also showed a control effect against *F. oxysporum* infected red pepper, strawberry, and tomato in the in vivo pot test. Genome mining of *S. collinus* Inha504 revealed the presence of the biosynthetic gene cluster (BGC) in the chromosome encoding a polyene macrolide which is highly homologous to the lucensomycin (LCM), a compound known for effective in crop disease control. Through genetic confirmation and bioassays, the antifungal activity of *S. collinus* Inha504 was attributed to the presence of LCM BGC in the chromosome. These results could serve as an effective strategy to select novel *Streptomyces* strains with valuable biological activity through bioassay-based screening and identify biosynthetic gene clusters responsible for the metabolites using genome mining approach.

## Introduction

Chemical fungicides have traditionally been employed for managing phytopathogenic fungi like *Fusarium*, *Botrytis*, and *Colletotrichum*, all of which pose substantial risks to crop yields in conventional agriculture^1,2^. However, the use of chemical fungicides has restricted due to worries related to their human health impacts and environmental contamination^3^. As a result, alternative methods are being explored to inhibit these phytopathogenic fungi that damage crops without having these adverse effects. One promising approach is to utilize soil-dwelling microorganisms, such as *Pseudomonas*, *Bacillus*, and *Streptomyces* species as eco-friendly pesticides^4^. Results of previous studies and commercial products containing these microbial strains have demonstrated significant potential for eco-friendly alternative pesticides as biocontrol agents against phytopathogenic fungi^5,6^.

Actinomycetes, primarily composed of *Streptomyces* species, are filamentous high G + C soil microorganisms renowned for their unique morphological differentiation and exceptional capacity to produce valuable bioactive compounds as secondary metabolites^7^. Extensive research has been conducted on these species to discover natural products with potential applications in both medicine and agriculture, as well as to develop novel derivatives of a natural product that is already known^7,8^. From the medicine aspect, natural products derived from actinomycetes are continuously being studied as potential sources such as antibiotic, anticancer, antiviral, and antioxidant drugs^9,10^. As the emergence of drug-resistant pathogens continues to present a growing threat, diverse strategies are currently being explored to uncover and systematically re-engineer novel and cryptic biosynthetic gene clusters (BGCs) within actinomycetes for the purpose of developing more advanced drugs^11^. From the agriculture aspect, the development of eco-friendly pesticides based on *Streptomyces* species has garnered considerable attention, as it offers a means of replacing the use of harmful chemical pesticides that cause environmental pollution^12^. Furthermore, *Streptomyces* continues to be reported in the soil environment, especially in the rhizosphere, to promote plant growth and control various diseases of plants through interaction with plants^13^.

Among the secondary metabolites biosynthesized by *Streptomyces*, polyenes, known for their strong antifungal activity, is typically characterized by having the polyketide core and 3 to 8 conjugated double bonds^14^. The polyene core’s synthesis is carried out by the enzymatic complex known as polyketide synthase (PKS) consisting of domains with various functions^15^. In addition, the polyene core is undergone to post-PKS modification by various enzymes including cytochrome P450, sulfonyl transferases, and glycosyl transferases^15^. Among the antifungal polyenes, macrocyclic polyenes such as amphotericin B, nystatin A1, natamycin and lucensomycin have been widely used to control fungi, such as being used to treat severe fungal infections, as a preservative in food, or to control fungal diseases in crops^14,16,17^. In addition, linear aminopolyol polyenes with amino or guanidino moieties such as linearmycin and mediomycin have potential to be applied as a wide range of antibiotics to have antifungal activity as well as antibacterial activity^18,19^. The antifungal mechanism of action of these polyene compounds hinges on the creation of ion channels, facilitated by their interaction with fungal ergosterol^20^. This interaction culminates in the permeabilization of fungal cell membranes, leading to the efflux of crucial intracellular ions, such as K^+^ and Mg^2+^, ultimately resulting in the demise of fungal cells^20^.

**Figure 1:**
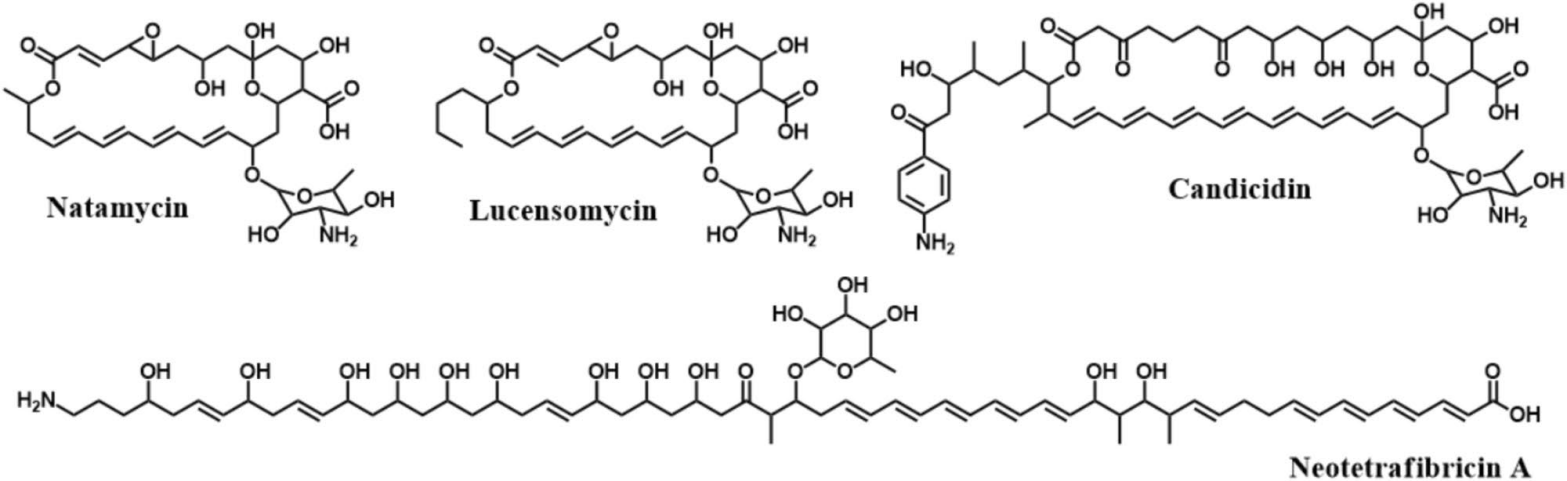
Actinomycete-derived various polyene compounds against phytopathogenic fungi.

Thus far, several polyenes have been derived from *Streptomyces* as active ingredients for controlling diseases caused by various phytopathogenic fungi (Fig. 1). Natamycin, a polyene macrolide compound biosynthesized by *S. chattanoogensis* L10, has been reported to exhibit inhibitory effects against a spectrum of phytopathogenic fungi. Notably, it impedes the growth of *Penicillium digitatum*, *Rhizopus stolonifer*, *Alternaria alternata*, and *Botrytis cinerea*, causative agents of fruit decay, black bread mold, leaf spots, and gray mold, respectively^17^. In a similar vein, Lucensomycin, another polyene macrolide compound synthesized by *S. plumbeus* strain CA5, has demonstrated its promise in inhibiting the growth of *Botrytis cinerea*, responsible for causing gray mold^21^. Furthermore, neotetrafibricin A, a linear aminopolyol polyene compound produced by *S. rubrisoli* Inha 501, has showcased its effectiveness in restraining the growth of a diverse array of phytopathogenic fungi. These include *Aspergillus niger*, *Alternaria alternata*, *Colletotrichum gloeosporioides*, and various *Fusarium* strains, implicated in black mold, leaf spots, anthracnose, and fusarium wilt, respectively^19^. Collectively, these findings underscore the potent antifungal capabilities of *Streptomyces* strains, producers of polyenes as secondary metabolites, in effectively controlling a wide spectrum of phytopathogenic fungi.

In a previous study, 149 antifungal strains were selected by conducting antifungal bioassay of about 2400 actinomycetes. Also, HPLC analysis confirmed that 51 strains among the selected cultures biosynthesized polyene compounds. Among these strains, one strain showing the strongest antifungal activity could produce polyene compounds and was highly homologous to *Streptomyces collinus* (named *Streptomyces collinus* Inha504). The in vitro bioassay and in vivo pot test against phytopathogenic fungi confirmed that *S. collinus* Inha504 could be a promising candidate, providing an alternative and eco-friendly solution for controlling phytopathogenic fungi in agriculture.

## Materials and methods

### Strains and growth conditions

*Streptomyces collinus* Inha504 was distributed from the Industrial Biomaterial Research Center, Korea Research Institute of Bioscience and Biotechnology (KRIBB), South Korea. *S. collinus* Inha504 was grown in BD™ tryptic soy broth (TSB) at 30 °C for the seed culture and BD Difco™ ISP4 agar at 30 °C for the sporulation. Conjugants were grown on mISP4 (BD Difco™ ISP4 37 g, tryptone 1.5 g, and yeast extract 0.5 g per liter) at 30 °C. All *Escherichia coli* strains were incubated at 37 °C in LB (tryptone 10 g, yeast extract 5 g, NaCl 5 g, glucose 1 g, and agar 15 g per liter) medium (appropriate antibiotics added where necessary). *Alternaria alternata* KACC (Korean Agricultural Culture Collection) 40,019, *Aspergillus niger* ATCC (American Type Culture Collection) 9642, *Candida albicans* ATCC 14,053, *Colletotrichum gloeosporioides* KACC 40,003, *Fusarium oxysporum* KACC 42,795, *Fusarium solani* KACC 44,891, *Fusarium semitectum* KCTC (Korean Collection for Type Cultures) 16,672, and *Fusarium verticillioides* KCTC 6065 were grown on PDA medium (potato starch 4 g, glucose 2 g, and agar 15 g per liter) at 28 °C for 3 days.

### Antifungal pot test

For in vivo antifungal activity against Fusarium wilt, *S. collinus* Inha504 was cultured in TSB medium for 3 days. To prepare red pepper, strawberry, and tomato seedlings for in vivo pot test, the crop seedlings were grown in vinyl pots (diameter 10 cm) at 31.2 ± 1 °C for 2 weeks. Five tree seedlings were treated with 100 mL of the 500-fold diluted *S. collinus* Inha504 culture medium (about 10^6^ CFU/mL), and a negative control was treated only with 100 mL of TSB medium. After 24 h, the potted seedlings were wounded on the stem and treated with *Fusarium oxysporum* KACC 40,051 cultured in PDB medium for 7 days. After 2 weeks, the appearance of the seedlings was examined to measure the degree of Fusarium wilt infection (0: disease-free–100: crop failure). The pot-to-pot significance difference test was performed at the 95% level using the DMRT method^22^.

### Genome sequencing of *S. collinus* Inha504

The *S. collinus* Inha504 genome was sequenced at Macrogen (South Korea) using the Illumina HiSeq (Illumina, USA) platforms and the PacBio RSII (Pacific Biosciences, USA). Library preparation for Illumina HiSeq sequencing was performed using the TruSeq DNA sample preparation kit for Illumina (NE, USA), with a library insert size of 350 bp; Library preparation for PacBio RS SMRT sequencing was performed using the PacBio DNA Template Prep Kit 1.0 (Pacific Biosciences, USA) and the library insert size was 20 kb. A high-quality sequence was obtained by correcting the assembled contig error using Pilon (v1.21) software. The de novo assembly of the sequenced fragments was performed using HGAP (v3.0) performs. The validation check of the analyzed fragments was performed using BLAST (v2.7.1 +) software and BUSCO (v3.0) software. The annotation was performed using Prokka (v1.12b) software.

### Production and purification of lucensomycin

*S. collinus* Inha504 was inoculated in 200 mL of TSB medium at 30 °C and 220 rpm for 48 h. The pre-cultures were added to 2 L of GSS medium (soybean flour 25 g, glucose 20 g, soluble starch 10 g, yeast extract 4 g, CaCO_3_ 2 g, NaCl 2 g, beef extract 1 g, and K_2_HPO_4_ 0.25 g per liter) in a 5 L bioreactor for batch fermentation. After 120 h cultivation, the culture broth was extracted 1:1 with *n*-butanol and concentrated using a vacuum evaporator. The concentrated extract was dissolved in methanol, loaded onto a column packed with a C-18 reversed-phase silica gel (Daiso, Japan), and eluted with methanol–water (30:70, v/v) to remove the residual medium components from the GSS medium. The eluted extract was purified using the fraction collector (Interchim, France) on a gradient comprised of solvents A (water) and B (methanol): 0–10 min, 30% B (v/v); 10–110 min 30–100% B; and 110–120 min 100% B at a flow rate of 20 mL/min^23^. The fractions containing lucensomycin with > 90% purity were detected at 303 nm and analyzed by HPLC. For the chromatographic conditions, solvent A (50 mM ammonium acetate, pH 6.5) and solvent B (methanol) were used for elution and loaded onto ZORBAX SB-C18 (4.6 mm × 250 mm, 5 μm, Agilent, USA). The column was equilibrated with 50% B (v/v); the flow rate was set to 1.0 mL/min using the following conditions: 0–3 min, 50–75% B; 3–30 min 75–100% B; 30–33 min 100–50% B; and 33–40 min 50% B^23^.

### LC–MS analysis

The lucensomycin showing > 90% purity was analyzed using Waters Synapt™ XS Q-ToF HDMS (Waters, Milford, CT, USA) coupled with Waters UPLC I-class (Waters, Milford, CT, USA). Mass spectrometry was equipped electrospray ionization (ESI) source and analysis was conducted using MS mode. The scan mass range was set from m/z 50 to 2500, and the scans were set as 1 s duration. Detailed ionization condition and MS method was as follow: for ionization, positive and negative capillary voltage were 3.0 kV and 2.5 kV, MS source temperature was 150 °C in case of positive mode and 100 °C in negative mode, desolvation gas temperature was 500 °C in case of positive mode and 250 °C in negative mode with 600 L/h gas flow. For detection, MS method was set to sensitivity mode and sampling cone voltage was 40 V, and detector voltage was set to 2250 V. MS machine was calibrated before detection using sodium iodide calibrant, and all analyzing process were revised using leucine encephalin in real time. For the chromatographic conditions, mobile phase A (0.1% formic acid in distilled water) and B (0.1% formic acid in acetonitrile) were used for elution and prepared sample was applied onto a Waters BEH C18 UPLC column (2.1 mm × 150 mm, 1.7 μm). The flow rate was set to 0.2 mL/min under the following conditions: 0–1 min, 80% A; 1–3 min, 80–60% A; 3–13 min, 60–30% A; 13–15 min, 30–10% A; 15–17 min, 10% A; 17–18 min 10–80% and 18–20 min, 80% A.

### Inactivation of LCM S3 gene

An *LCM S3* gene inactivation cassette, including a homologous arm of the *LCM S3* gene, was constructed by PCR amplification using the following primer pairs: LCM_S3_del._F (5′- GCCAGTGCCAAGCTTGTCTACACCCAAGGACAGC-3′) and LCM_S3_del._R (5′-CTGCAGCCCAAGCTTAGTCACCGCCGTCGTAGAA-3′) (Fig. S5). The amplified fragments were ligated into pKC1132 digested with *Hind*III using an In-Fusion Cloning kit (Takara Bio, Japan). The *LCM S3* gene inactivation cassette was then introduced to *E. coli* ET12567/pUZ8002 and conjugated directly with *S. collinus* Inha504 by homologous recombination. The *LCM S3* disrupted mutants were selected on ISP4 agar plate containing apramycin, and then PCRs were performed to verify its genotypes.

### In vitro assays for biological activities

The in vitro antifungal assay was performed based on The Clinical and Laboratory Standards Institute document M27-A3^24^. After *C. albicans* was cultured in PDB medium at 30 °C for 1 day, the cultured solution was diluted with PDB medium until the OD value reached 0.3 at 530 nm. A working suspension was prepared with a 1:2000 dilution with RPMI-1640 broth media (with glutamine and phenol red, without bicarbonate, Sigma–Aldrich, USA), resulting in 5.0 × 10^2^–2.5 × 10^3^ cells per μL. Various concentrations (25–1600 μg/mL) of polyene antibiotics dissolved in 10 μL DMSO were added to the working suspension of 990 μL. The mixtures were then incubated at 30 °C without shaking for 2 days. The colorimetric red-to-yellow change in the mixture indicated the growth of *C. albicans.* The minimum inhibitory concentration (MIC) was determined by measuring the minimum concentration that changed the color to yellow. The experiment was performed in duplicate^19^.

The in vitro hemolysis assay was performed using a previously reported method^25^. Defibrinated horse blood was purchased from Kisan Biotech (South Korea). The polyene compounds were diluted to 1–200 μg/mL with DMSO. A 50 μL sample of each polyene solution was added to 450 μL of 2.5% defibrinated horse blood buffered with RBC buffer (150 mM NaCl, 10 mM NaH_2_PO_4_, 1 mM MgCl_2_, and pH 7.4), resulting in a tenfold dilution of each concentration of polyene. The samples were then incubated at 37 °C for 30 min. After incubation, the samples were centrifuged at 10,000 g for 2 min. Subsequently, 100 μL of the supernatant from each sample was added to a 96-well plate, and the absorbance was read at 540 nm using a microplate reader (TECAN, Switzerland). The percentage hemolysis of each sample was defined as (Abs_sample_−Abs_negative_/Abs_positive_−Abs_negative_) × 100 to calculate the Minimum Hemolysis Concentration (MHC)^26^.

### Ethical approval and consent to participate

All methods were carried out in accordance with relevant guidelines and regulations in this paper.

## Results

### Isolation of the *S. collinus* Inha504 produced antifungal polyene compounds

**Figure 2:**
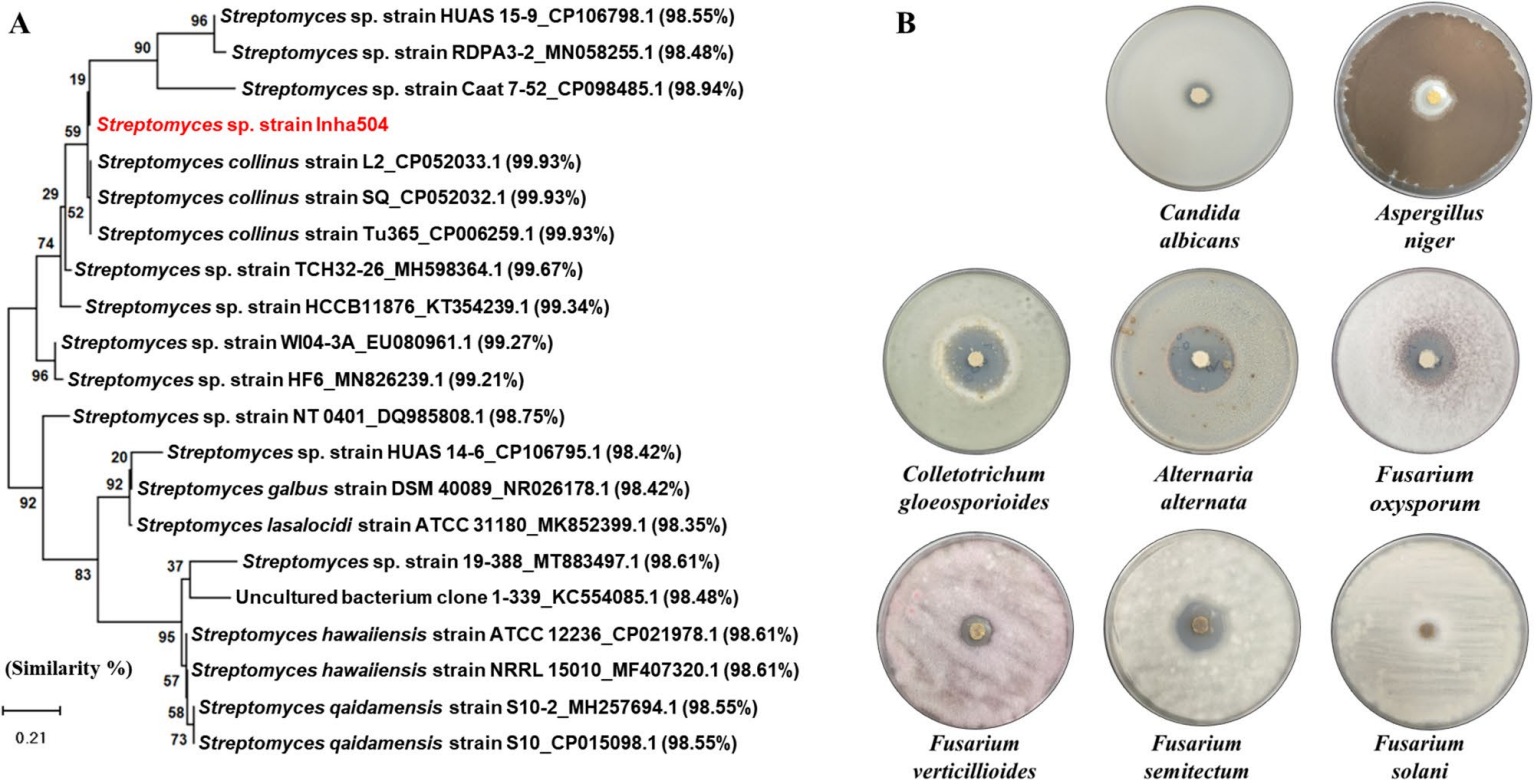
(**A**) Phylogenetic tree of *S. collinus* Inha504 based on the 16S rRNA sequence; the evolutionary history was inferred using the Neighbor-Joining method. Evolutionary analyses were conducted in MEGA7^33^ (**B**) Antifungal assays of *S. collinus* Inha504 against various phytopathogenic fungi.

Through previous studies, 51 strains with antifungal activity and producing polyene compounds were screened among 2419 actinomycetes^19^(Fig. S1). Among the screened strains, one *Streptomyces* sp., showing strong antifungal activity against *C. albicans* and *F. oxysporum*, was selected as the final candidate for the development of eco-friendly pesticides based on actinomycetes. The final selected strain was isolated from the organic green tea fields in Jeolla Province, South Korea (34°51′31.7″N 127°08′48.1″E) and showed 99.93% similarity to *Streptomyces collinus* strains by phylogenetic analysis based on 16S rRNA sequences (Fig. 2A). The final selected strain was named *Streptomyces collinus* Inha504.

### Antifungal bioassay of *S. collinus* Inha504 against various phytopathogenic fungi

In order to confirm the potential of *S. collinus* Inha504 to develop into a microorganism based eco-friendly pesticide, antifungal bioassay was performed using agar plugs cultured for 7 days on GSS plate against various phytopathogenic fungi, *Fusarium oxysporum, Fusarium solani*, *Fusarium verticilliodes*, *Fusarium semitectum*, *Aspergillus niger, Colletotrichum gloeosporioides*, and *Alternaria alternata* (Fig. 2B). As shown in Fig. 2B, *S. collinus* Inha504, which had clear inhibition zones around the agar plug of the strain, was confirmed to inhibit the growth of the most phytopathogenic fungi tested.

**Figure 3:**
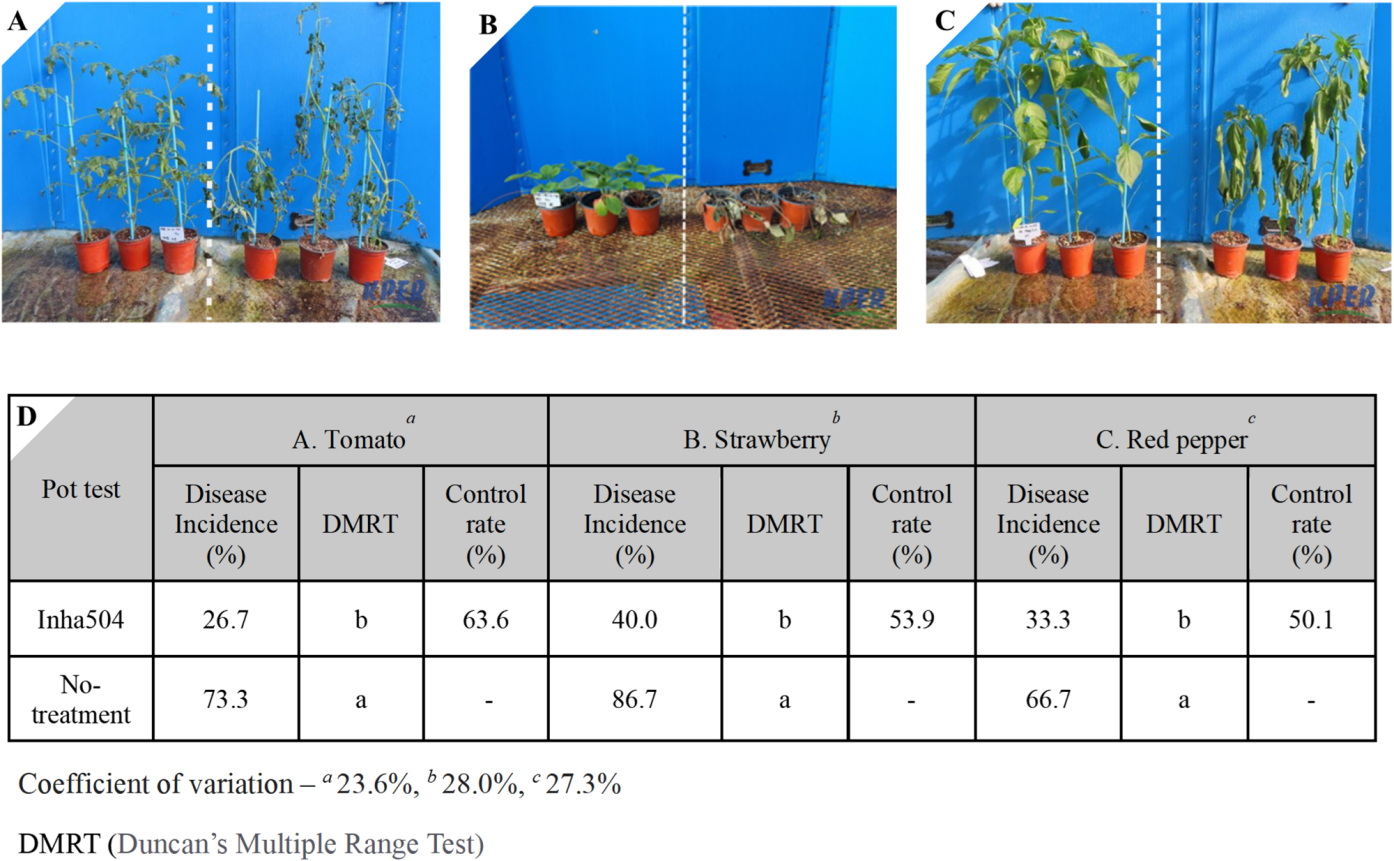
In vivo pot test of *S. collinus* Inha504 against *F. oxysporum*. A pot test was conducted on (**A**) tomato, (**B**) strawberry, and (**C**) red pepper. The left side of (**A**), (**B**), and (**C**) was treated with 500-fold diluted *S. collinus* Inha504 culture medium, and the right side was treated with TSB medium (negative control). (**D**) The control rate of *S. collinus* Inha504 for each crop.

Also, several crops were infected with *F. oxysporum* (KACC 40,051), which causes wilt disease in plants, and treated with *S. collinus* Inha504 culture broth to confirm whether the strain could be applied as microbial fungicide through in vivo pot-scale test (Fig. 3). The crops used in the pot-scale test were red pepper, strawberry, and tomato, and the control rates were 50.1%, 53.9%, and 63.6%, respectively, and the control rate was converted based on the infection rate by repeating five seedlings three time (Figs. 3 and S2). Through pot-scale test, Inha504 has been proven to have good potential as microbial fungicide, with the control rate of more than 50% that can be registered as an organic agricultural material in Korea.

### Bioinformatic analysis of *S. collinus* Inha504 whole genome sequence

**Figure 4:**
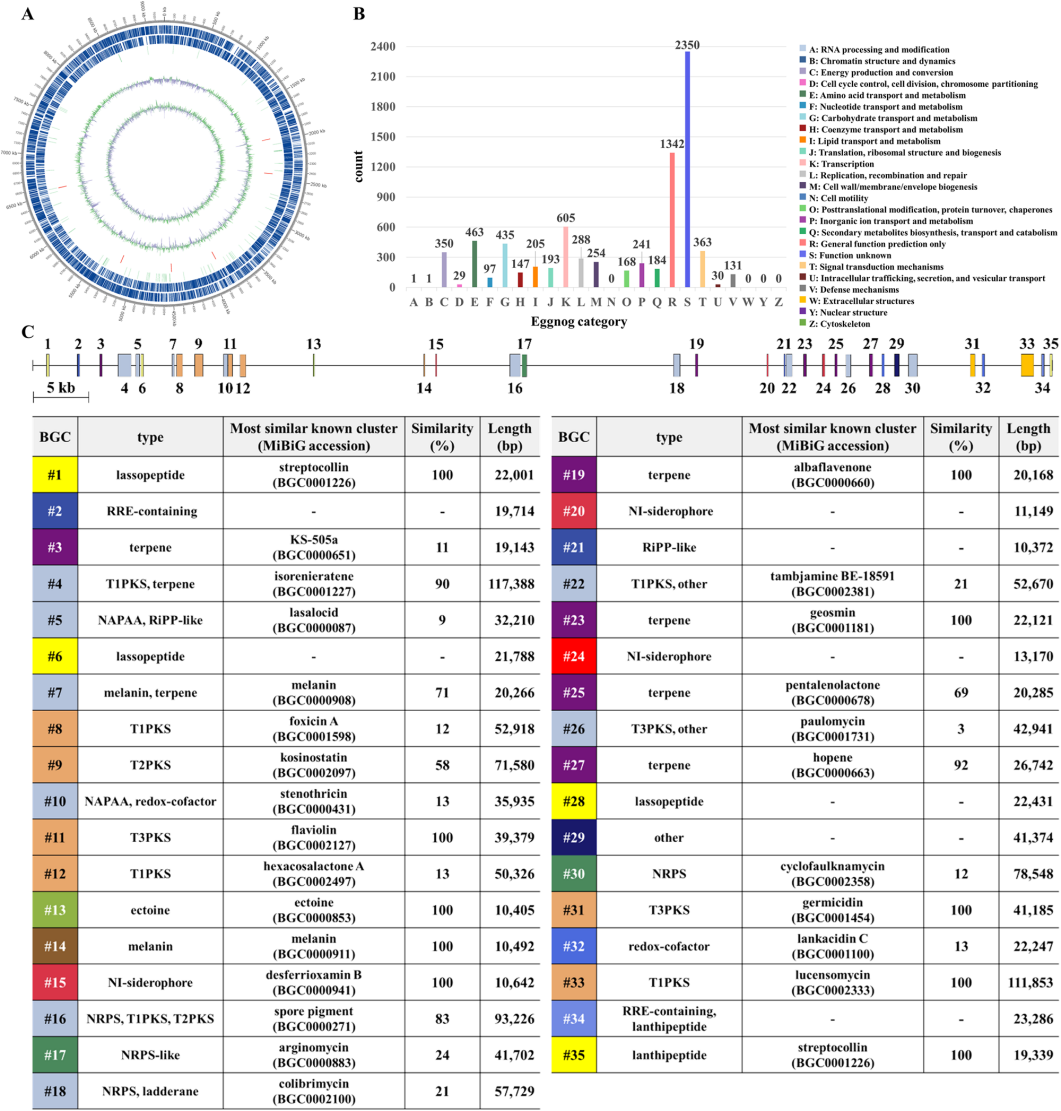
(**A**) Circular whole-genome map drawn by applying the annotation result of the *S. collinus* Inha504 chromosome. Marked characteristics are shown from the outside to the center; CDS on the forward strand, CDS on the reverse strand, tRNA, rRNA, GC content, and GC skew (**B**) Classification of CDS by eggnog annotation (**C**) Analysis of secondary metabolite biosynthetic gene clusters by antiSMASH 7.0

Whole-genome sequencing of *S. collinus* Inha504 was performed preferentially to identify the biosynthetic gene cluster (BGC) present on the chromosome responsible for the production of target polyene macrolide compounds. The complete genome sequence was deposited in the National Center for Biotechnology Information (NCBI) with an accession number NZ_CP136667.1. The whole genome size was 9,194,571 bp, no plasmid was found, and the GC content was 72.07% (Fig. 4A, Table S1). In addition, the genome contained 8072 genes, 18 rRNAs, and 90 tRNAs (Table S1). The encoding gene sequences were aligned with the eggnog database to predict the estimated gene function, of which 7797 genes, 96.6% of the total genes, were successfully annotated by Eggnog databases^27^(Fig. 4B).

*S. collinus* Inha504 whole genome sequence was analyzed to reveal the BGCs of the secondary metabolites associated with antifungal activity by antiSMASH. By the antiSMASH 7.0, *S. collinus* Inha504 chromosome contains 37 tentative BGCs of the secondary metabolites^28^(Fig. 4C). In detail, there were 15 clusters associated with polyketide synthase (PKS) and non-ribosomal peptide synthetase (NRPS), which are well known for condensing using small building blocks to produce larger natural products, including four T1PKSs, one T2PKS, three T3PKSs, five NRPSs, and two NRPS-PKS hybrid clusters. The other clusters were analyzed to belong to five terpenes, four lanthipeptides, three siderophores, two melanins, one lassopeptide, and one ectoin cluster (Fig. 4C). Especially, BGC # 35 was predicted to be the most likely BGC to biosynthesize the target polyene macrolide compound, lucencomycin (LCM).

### Identification of the BGC corresponding to the antifungal compound in *S. collinus* Inha504 chromosome

**Figure 5:**
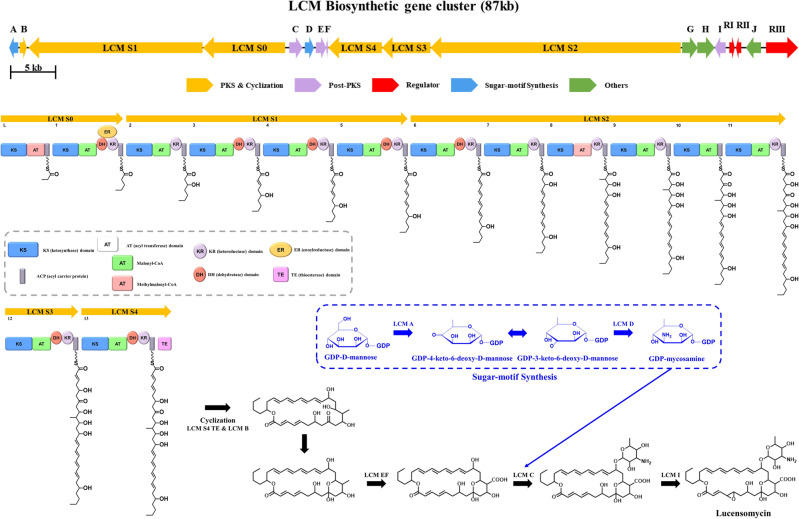
ORFs encoded in the putative LCM biosynthetic gene cluster and proposed biosynthetic pathway to LCM (more details of ORFs are listed in Table 1). The light gray dash line represent the domains of polyketide synthases (PKSs) in LCM BGC, and the blue dash line is the sugar motif (rhamnose) synthesis (Created with BioRender.com).

Based on previous studies of LCM and bioinformatic analysis of BGC #35, the polyene macrolide biosynthesized by *S. collinus* Inha504 was judged as LCM, and we intend to prove this judgment^29^(Figs. 4C and S3). First, the biosynthetic pathway by BGC #35 was predicted based on bioinformatic analysis, and the structure of the predicted compound was confirmed.^29^(Fig. 5, Table 1). And then, the LC–MS analysis of *S. collinus* Inha504 cultured and purified polyene compounds (> 90% purity) observed a signal of m/z 708.3626 for [M + H]^+^ and m/z 706.3430 for [M−H]^−^, which matched the calculated signal of m/z 708.3590 for [M + H]^+^ and m/z 706.3444 for [M−H]^−^ in LCM^29^(Fig. S4). Finally, we disrupted the *LCM S3* (PKS gene) in BGC #35 to confirm that BGC #35 corresponds to LCM (Fig. S5). As expected, HPLC analysis of *LCM S3*-disrupted mutant cultures confirmed that the LCM peak disappeared (Fig. S6). Therefore, BGC #35 is responsible for the biosynthesis of LCM in the *S. collinus* Inha504.

**Table 1:** Annotation of the ORFs in the LCM biosynthetic gene cluster.

ORF	Size^a^	Predicted function	Strand	*S. cyanogenus* S136 homolog^b^ (%)
LCM A	344	GDP-mannose 4,6-dehydratase	**−**	97
LCM B	256	Thioesterase	** + **	91
LCM S1	6442	Type I PKS	**−**	93
LCM S0	3033	Type I PKS	**−**	95
LCM C	483	Glycosyltransferase	** + **	95
LCM D	352	GDP-perosamine synthase	** + **	97
LCM E	392	Cytochrome P450	** + **	96
LCM F	64	Ferredoxin	**−**	91
LCM S4	1990	Type I PKS	**−**	96
LCM S3	1789	Type I PKS	**−**	94
LCM S2	9250	Type I PKS	**−**	95
LCM G	578	ABC transporter	** + **	95
LCM H	640	ABC transporter	** + **	96
LCM I	386	Cytochrome P450	**−**	93
LCM RI	192	Transcriptional regulator	** + **	91
LCM RII	200	Transcriptional regulator	**−**	93
LCM J	549	GMC oxidoreductase	**−**	96
LCM RIII	1186	Transcriptional regulator	** + **	91

^a^Size in the number of amino acids.

^b^*S. cyanogenus* S136 is the production strain of lucensomycin^29^. The homolog shows amino acid sequence identity (%).

### In vitro antifungal activity and hemolytic toxicity of LCM

**Figure 6:**
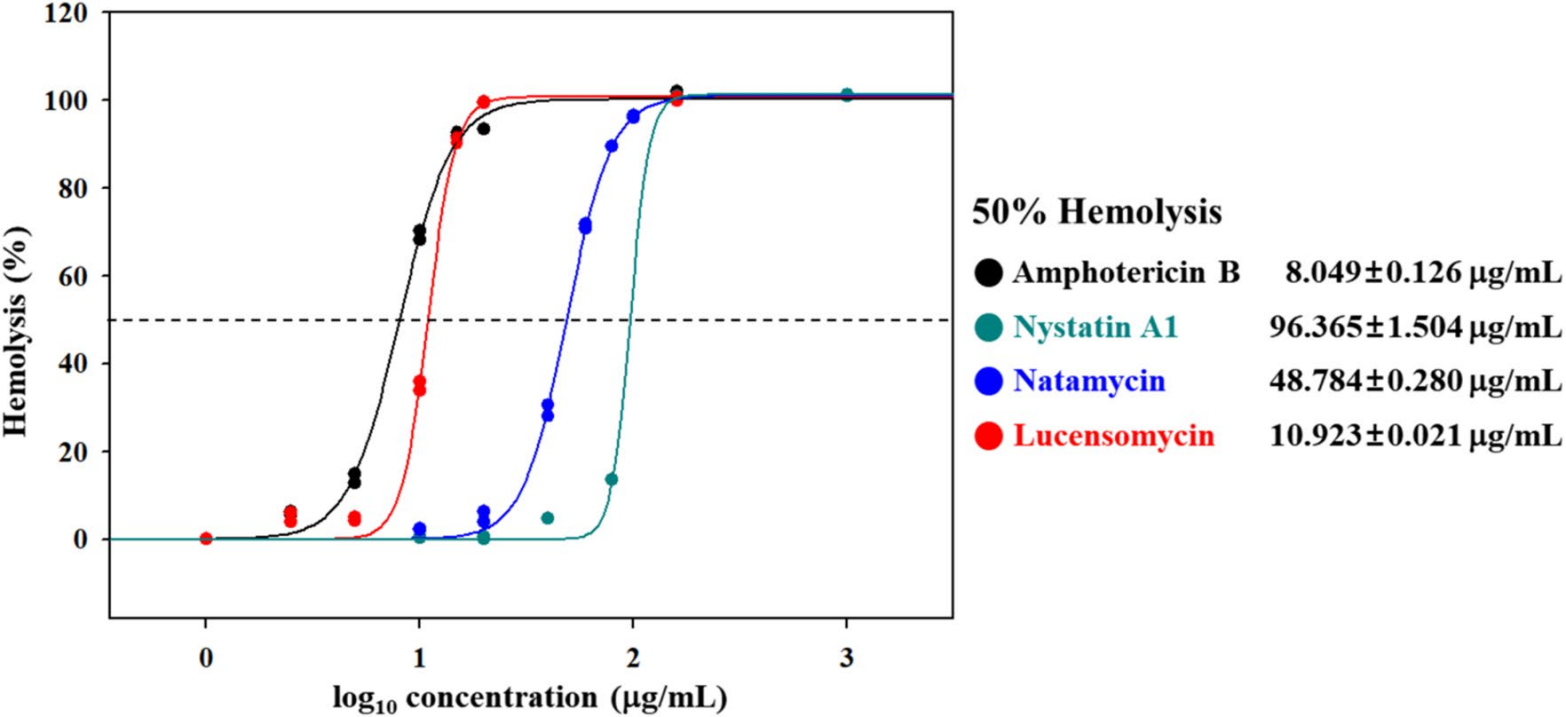
In vitro toxicity of polyene macrolides. The minimum hemolytic concentration (MHC) evaluation assays (values causing 50% hemolysis against horse blood cells percentage standard deviation).

The purified LCM was evaluated for both in vitro antifungal activity against *C. albicans* and the hemolytic toxicity with horse blood. The antifungal activity was measured by the minimum inhibitory concentration (MIC) evaluation assays using the colorimetric change in the RPMI-1640 media containing the fungus^24^. Interestingly, the MIC of LCM (0.25 μg/mL) against *C. albicans* was lower than amphotericin B (0.5 μg/mL) and natamycin (2 μg/mL) (Table 2, Fig. S7). Moreover, the MHC (minimum hemolytic concentration) value for LCM was 10.923 μg/mL, while for amphotericin B was 8.049 μg/mL. In contrast, the MHC value of natamycin was 48.784 μg/mL, indicating that the in vitro toxicity of LCM was lower than that of amphotericin B but higher than that of natamycin (Figs. 6 and S8). Based on in vitro assays for amphotericin B and LCM, it was confirmed that the antifungal activity of LCM against *C. albicans* was not only better than amphotericin B, but also lower hemolytic toxicity. Also, based on in vitro assays for natamycin and LCM, it is speculated that the difference in antifungal activity and toxicity may be attributed to the variation in the functional group at position C01.

**Table 2:** In vitro antifungal activity of polyene macrolides. MIC, minimum inhibitory concentration (values resulting in no visible growth of *C. albicans*).

	Amphotericin B	Nystatin A1	Natamycin	LCM
Antifungal activity (MIC, μg/mL)^a^				
*Candida albicans* ATCC 14,053	0.5	1	2	0.25

## Discussion

**Figure 7:**
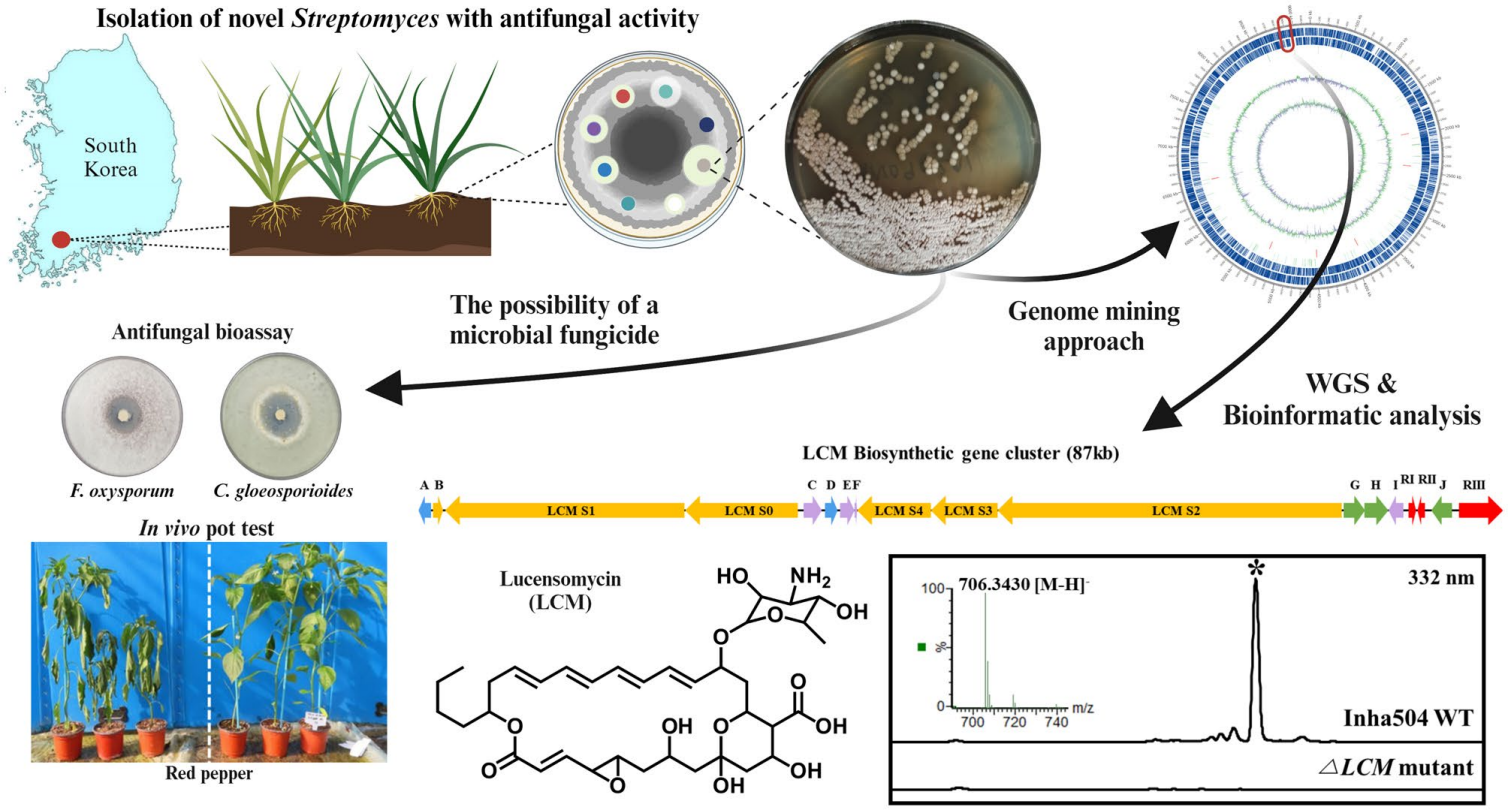
Isolation of novel *Streptomyces* having potential as bio-control agent and confirmation of LCM BGC (Created with BioRender.com).

Through this study using the antifungal activity-based screening approach, 149 strains of actinomycetes with antifungal activity were selected out of 2419 strains. Further HPLC analysis revealed 51 strains of actinomycetes capable of producing polyene compounds. On the other hand, the traditional activity-based screening approach encountered difficulties in uncovering the type and chemical structure of the target compound until structural analysis was conducted. To overcome these difficulties, 37 putative BGCs were predicted through the whole genome sequencing and BGC (gene cluster) analysis of *S. collinus* Inha504. Of these, the PKS genes contained ten BGCs as the core biosynthetic genes. Due to advances in sequencing technology and the accumulation of previous research on the biosynthetic pathway of natural products, predicting the biosynthetic pathway and structure of the type I PKS system has become increasingly straightforward, following the conventional rules of the PKS multi-domain modular system. Through antiSMASH analysis of all BGCs in *S. collinus* Inha504, BGC #35 encodes the target antifungal compound, which is the previously reported polyene macrolide known as lucensomycin (LCM)^29^. Gene disrupted experiment confirmed that BGC #35 is responsible for the presence of LCM. As a result, this research facilitated the efficient and predictable utilization of eco-friendly microorganisms as a promising alternative to synthetic fungicides with potential benefits for the nature and human health (Fig. 7).

This research confirmed the potential of eco-friendly microbial fungicides based on *Streptomyces* as alternatives to synthetic fungicides associated with various environmental and health issues. Several microbial fungicides based on *Streptomyces* strains are commercially available*,* such as AGBIO’s MYCOSTOP based on *S. griseoviridis* K61 producing the polyene macrolide candicidin, and NOVOZYMES’ ACTINOVATR based on *S. lydicus* WYEC108 producing the polyene macrolide natamycin^30^. Furthermore, ongoing research continue to confirm the potential of *Streptomyces* isolates as putative bio-fungicides. These include the discovery of *S. yongxingensis* JCM 34,965, which biosynthesizes niphimycin C for controlling banana fusarium wilt, the discovery of *S. rubrisoli* Inha501, which biosynthesizes I-NTF for inhibiting various phytopathogenic fungi, and the discovery of *Streptomyces* sp. FX13, which biosynthesizes oligomycin A for controlling fungicide-resistant *Botrytis cinerea*^19,31,32^.

The newly isolated *S. collinus* Inha504 inhibited various phytopathogenic fungi, highlighting its potential for applications as the biocontrol agent through in vivo pot test for crops (strawberry, tomato, and red pepper). To develop the microbial fungicide based on *S. collinus* Inha504, optimization of the culture condition and its formulation study is currently underway. Additionally, through genetic confirmation, it was demonstrated that the antifungal activity of *S. collinus* Inha504 was attributed to the presence of LCM BGC in the chromosome. These results provide an effective strategy to select a novel bacterial strain with valuable biological activity and identify a biosynthetic gene cluster responsible for the metabolite using a genome mining approach.

### Supplementary Information

Supplementary Information.

## Data Availability

The datasets used and/or analysed during the current study are available from the corresponding author on reasonable request.
