# Supplementary material for: Isolation of Streptomyces inhibiting multiple-phytopathogenic fungi and characterization of lucensomycin biosynthetic gene cluster
Source: Sci Rep. 2024 Apr 2;14:7757. doi: 10.1038/s41598-024-57888-0 (PMC10987574; doi:10.1038/s41598-024-57888-0)
Supplement: Supplementary file 1 — Supplementary Information. [file 41598_2024_57888_MOESM1_ESM.docx]

**Isolation of *Streptomyces* inhibiting multiple-phytopathogenic fungi and characterization of lucensomycin biosynthetic gene cluster**

**Heung-Soon Park, Seung-Hoon Kang, Si-Sun Choi, and Eung-Soo Kim^*^**

*Department of Biological Sciences and Bioengineering, Inha University, Incheon 22212, Republic of Korea*

^*^Corresponding authors: E-mail: eungsoo@inha.ac.kr; Phone: +82-32-860-8318; Fax: +82-32-865-4046

Key words: Actinomycetes, Antifungal activity, Phytopathogenic fungicide, Polyene macrolide, Genome mining

**Supplementary Fig. S1.** Screening to isolate the strains showing both antifungal activities against *C. albicans* and *F. oxysporum* and a typical polyene spectrum assayed by 2-dimensional HPLC analysis. (A) antifungal assay against *C. albicans* and *F. oxysporum* (B) HPLC analysis

**
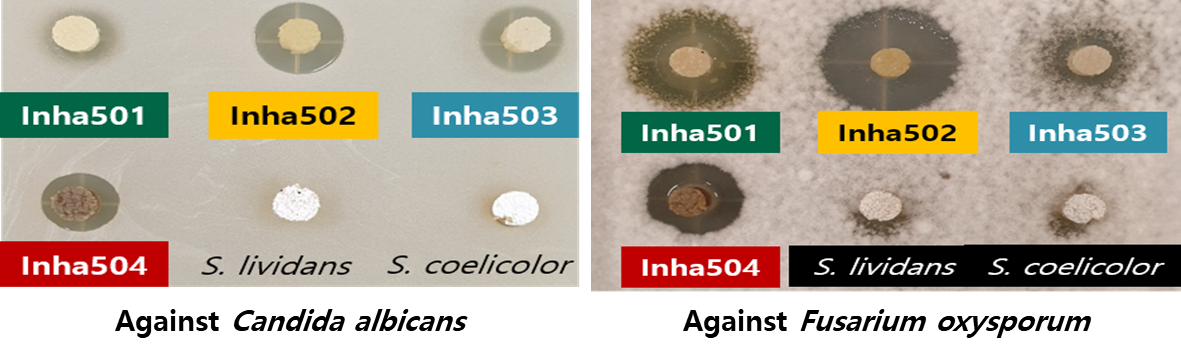
A.**

**
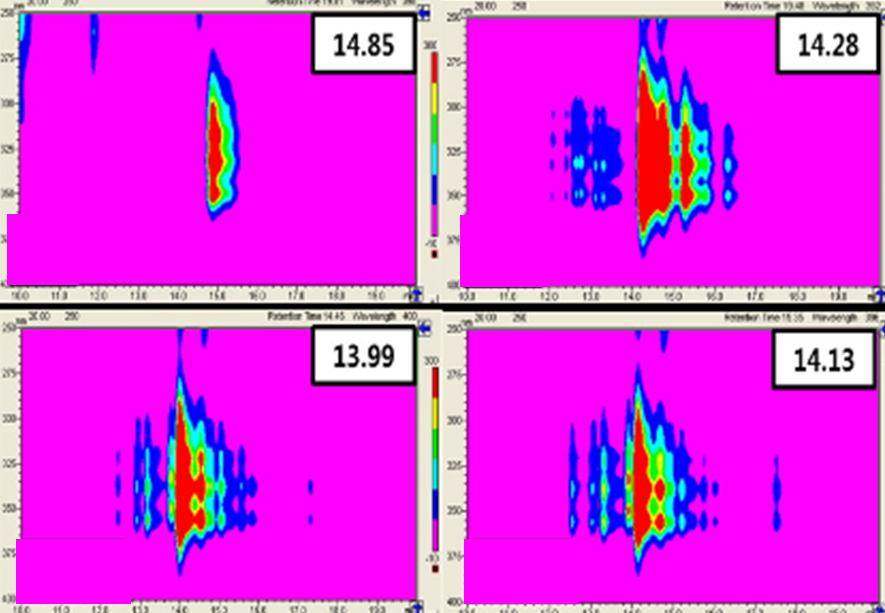
**

**B.**

**Supplementary Fig. S2.** *In vivo* pot test of *S. collinus* Inha504 against *F. oxysporum*


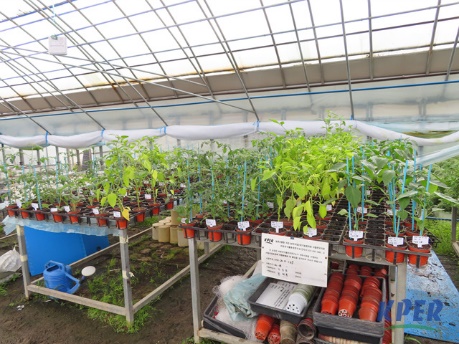

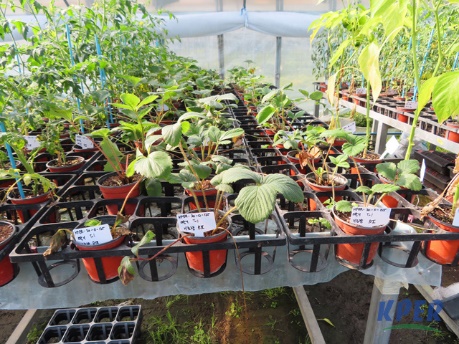


**< Panoramic view of the test area>**


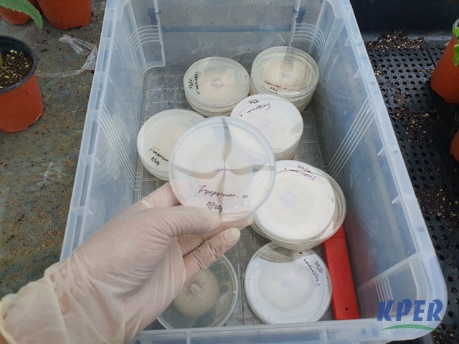

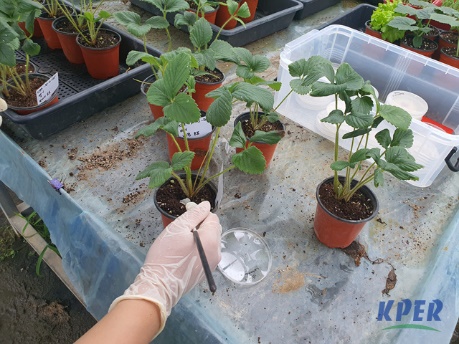


**< Pathogen inoculation>**


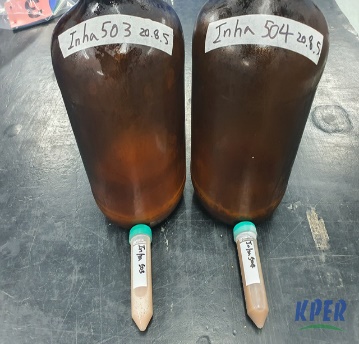

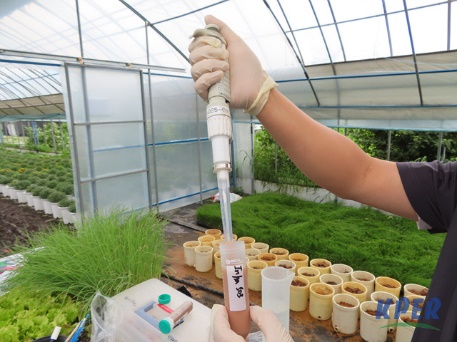

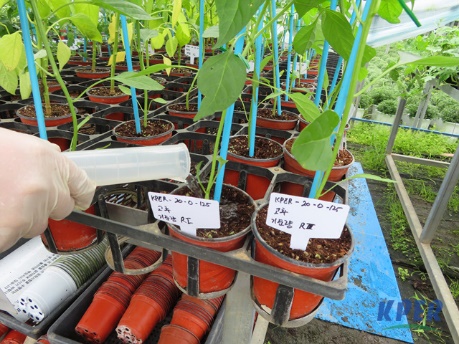


**< Culture broth treatment>**

**Supplementary Fig. S3.** Comparison of LCM BGCs in *S. collinus* Inha504 (LCM) and *S. cyanogenus* S136 (lcm) & Comparison of PKS domains in those BGCs

**
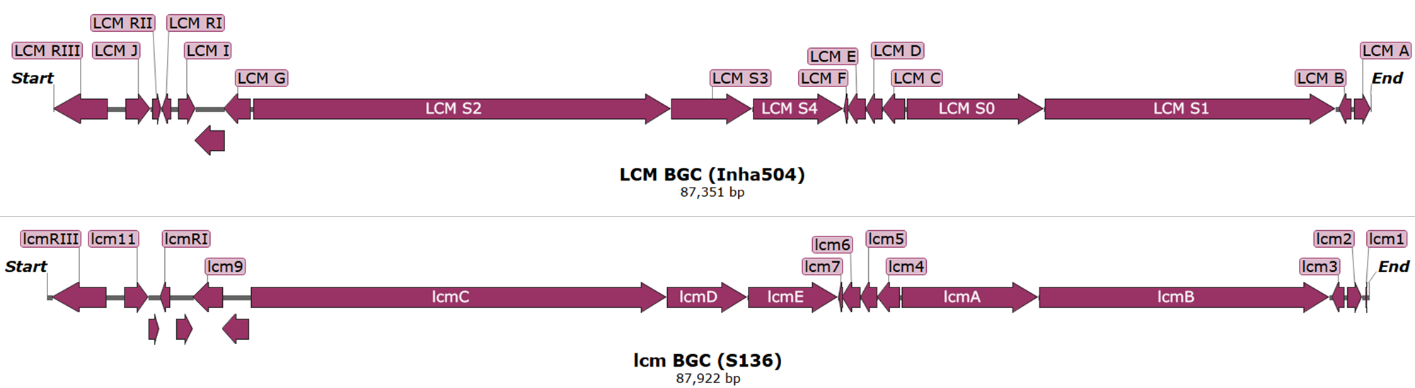
**


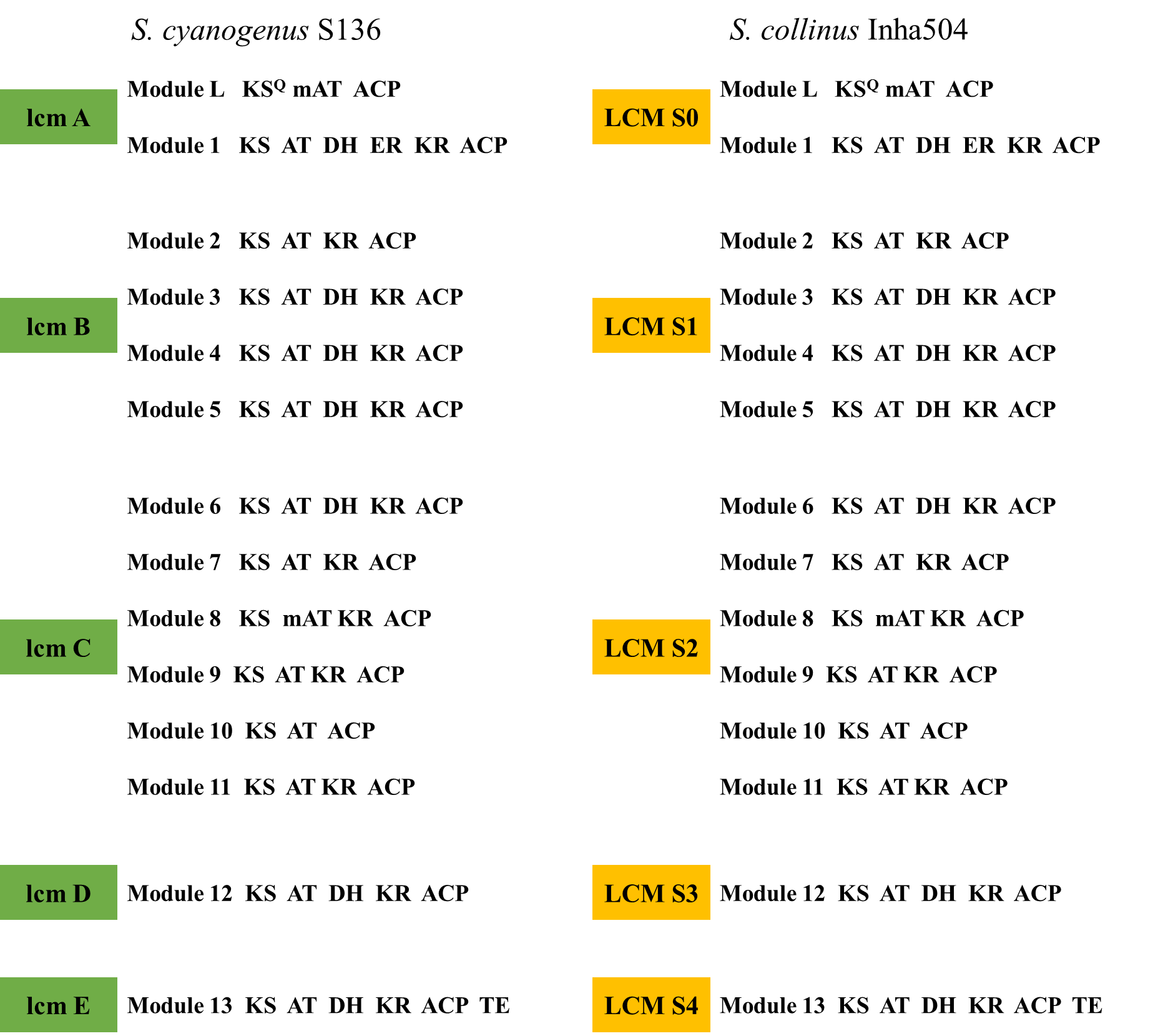


**Supplementary Fig. S4.** High Resolution Mass Spectrometry (HRMS) in positive and negative ion mode to measure the molecular weight of LCM.
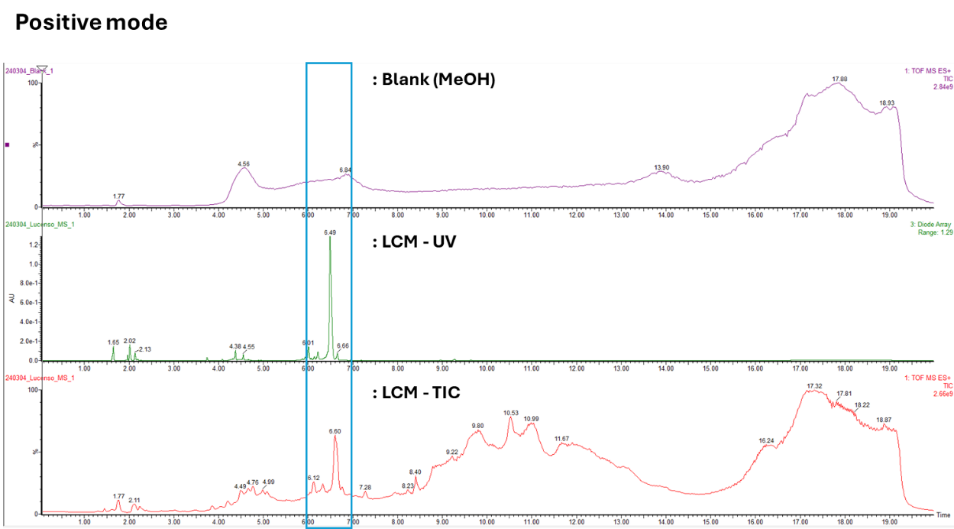


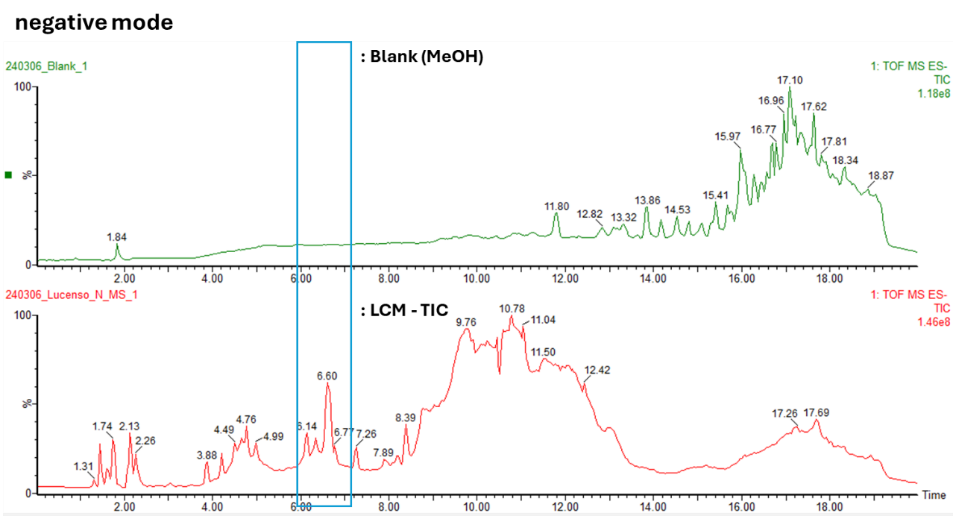


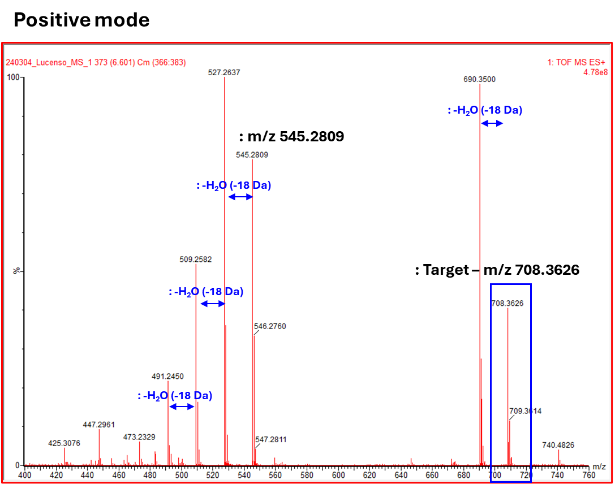

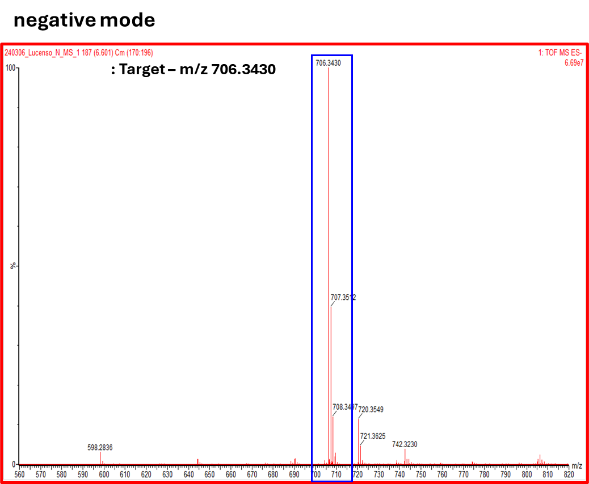


**Supplementary Fig. S5.** Confirmation of LCM BGC by gene knockout. (Check primer pair; F (5’- GTGAGATCGGCGTGCTGGAA-3’) and R (5’- TTCCGGCTCGTATGTTGTGT-3’)


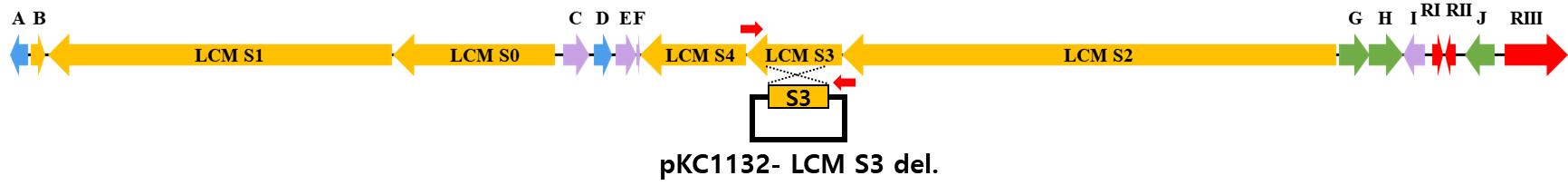


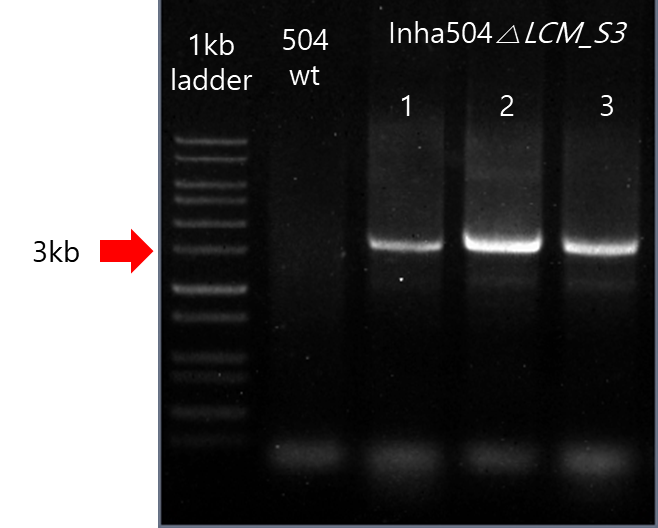


**Supplementary Fig. S6.** Comparison of HPLC Analysis Results of the wild-type *S. collinus* Inha504 (upper line) and *LCM S3* disrupted *S. collinus* Inha504 mutant (Inha504 *△LCM*, lower line) at 303 nm. The asterisked peak is confirmed to be the LCM and the small peak in front of LCM peak is believed to the derivative product of LCM.


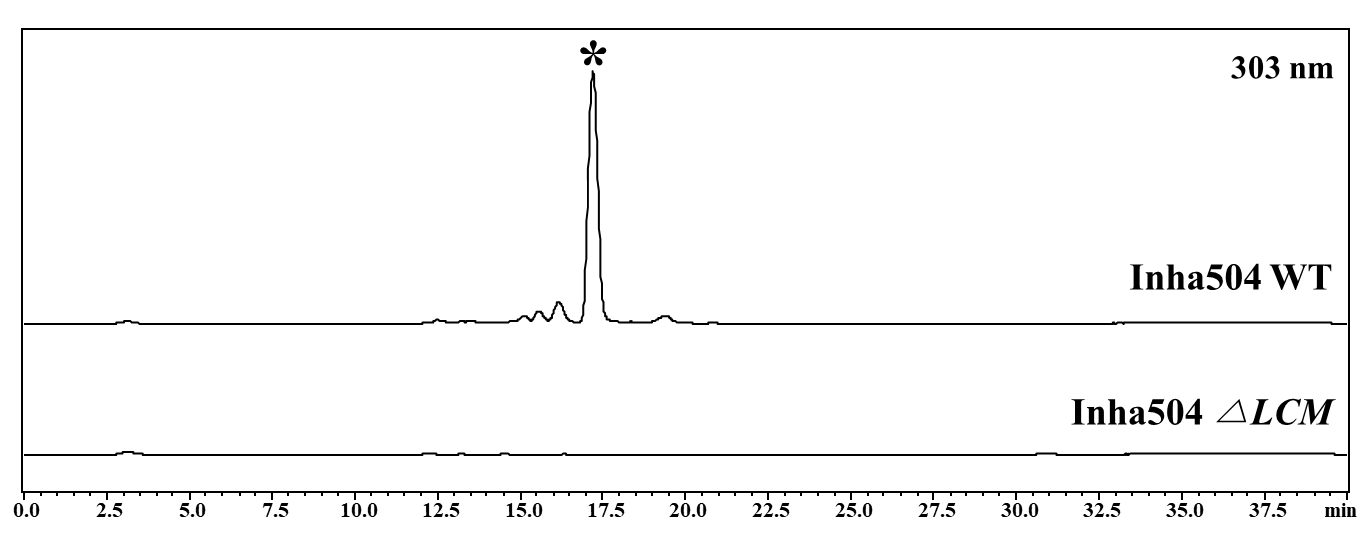


**Supplementary Fig. S7.** *In vitro* antifungal activity of LCM using the RPMI-1640. The minimum inhibitory concentration (MIC) values were determined by measuring the minimum concentration that changed color to yellow.


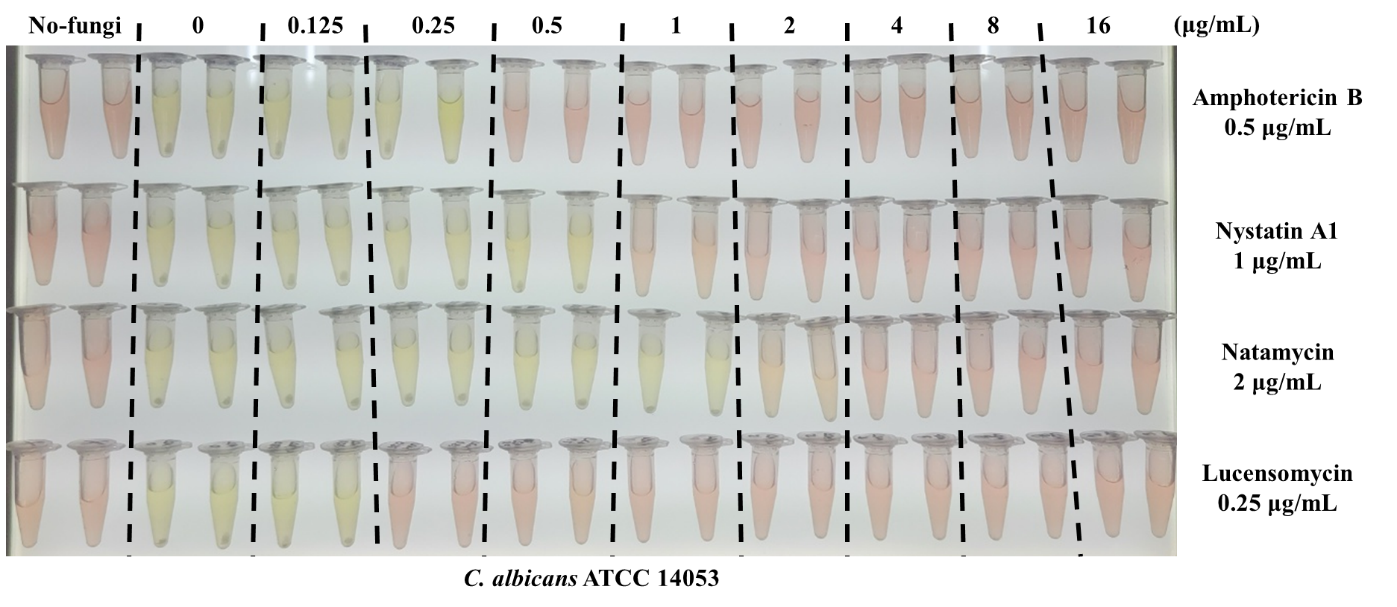


**Supplementary Fig. S8.** *In vitro* hemolytic toxicity of polyene macrolides.


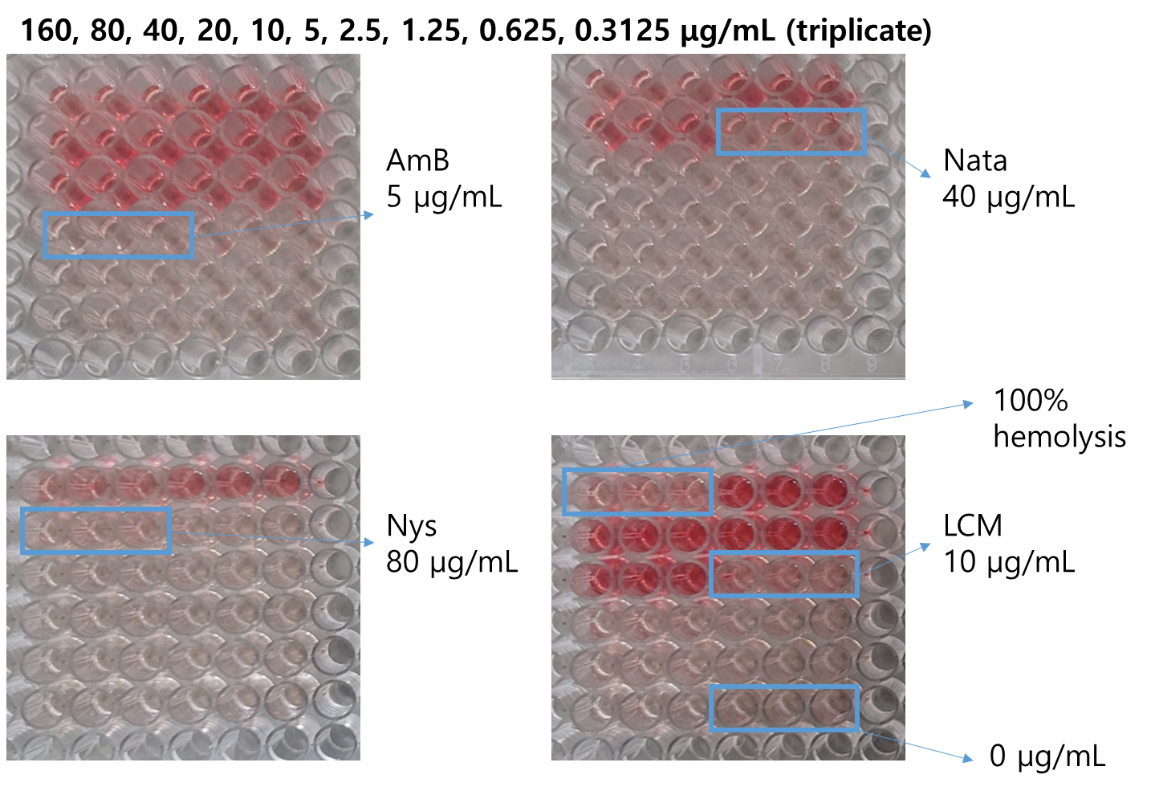


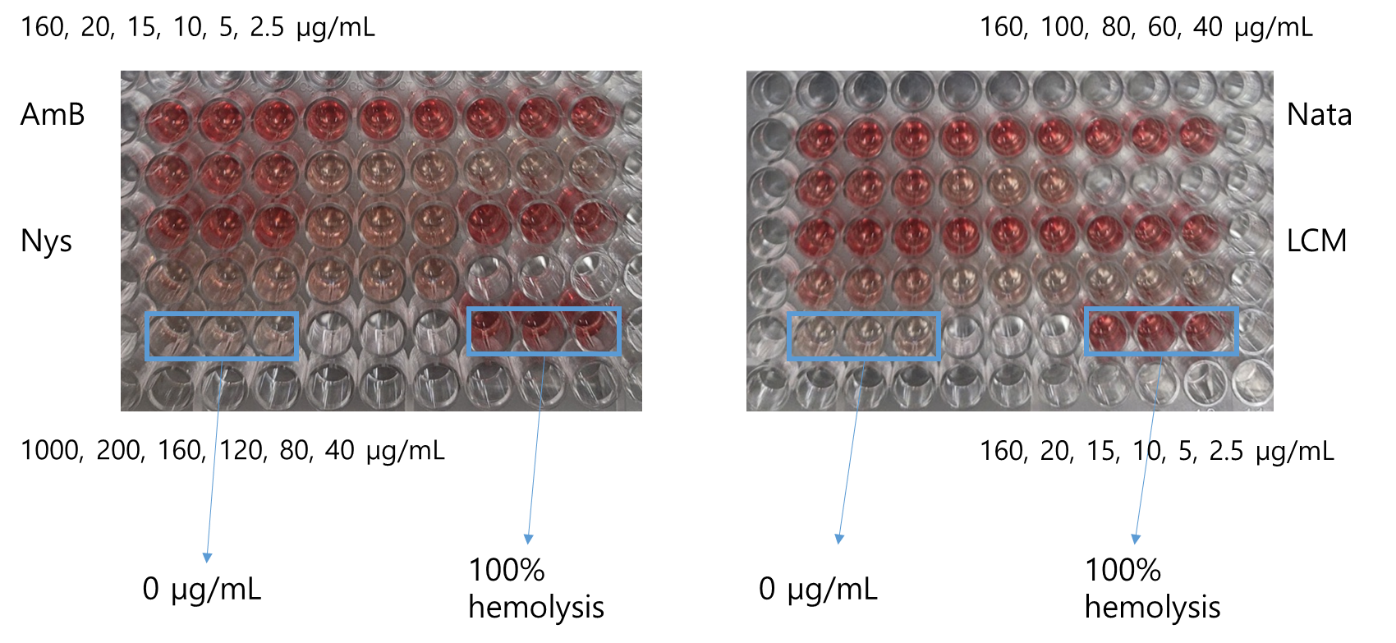


**Supplementary Table 1** Whole-genome sequencing of *S. collinus* Inha504

|  | Total read bases | Total reads | GC(%) | Q20 (%) | Q30 (%) |
| --- | --- | --- | --- | --- | --- |
| Raw dataset | **1,250,607,048** | **12,382,248** | **71.86** | **95.55** | **92.34** |
| Filtered dataset | **919,492,284** | **9,103,884** | **71.74** | **99.45** | **98.81** |

. Total Number of Bases: The Total number of bases sequenced.

. Number of Reads: The Total number of reads. In illumina paired-end data, read1 and read2 are added.

. GC (%): GC content

. Q20 (%): Ratio of bases that have phred quality score over 20.

. Q30 (%): Ratio of bases that have phred quality score over 30.

| Contig name | Length | GC (%) | Depth | Circular | Alias |
| --- | --- | --- | --- | --- | --- |
| contig1 | **9,194,571** | **72.07** | **91** | **NO** | **contig1** |
| Total | **9,194,571** | **72.07** | **91** |  |  |

. Length(bp): The number of bases in each contig

. GC (%): GC content

. Depth: The number of reads that overlap each contig

. Circular: 5’ end and 3’ end are connected.

. Alias: The alias is named based on the BLASTN(v2.7.1+) result.

The following two conditions are used to create an alias:

a. Query cover 80% or more

b. Similarity between genome size

| Contig name | Length | CDS | tRNA | rRNA |
| --- | --- | --- | --- | --- |
| contig1 | **9,194,571** | **8,072** | **90** | **18** |
| Total | **9,194,571** | **8,072** | **90** | **18** |

. Length(bp): The number of bases in each contig

. CDS: Coding sequences

. tRNA: Transfer RNA, tRNA has triplet nucleotide sequence complementary to the triplet nucleotide coding

sequences of messenger RNA. Circular: 5’ end and 3’ end are connected.

. rRNA: Ribosomal RNA, a molecular component of ribosome
